# Clinicopathological Aspects of Dilation and Curettage (D&C) Biopsies Taken from Patients Living at High Altitude in Taif, KSA, with a Special Emphasis on Chronic Endometritis

**DOI:** 10.3390/life14081021

**Published:** 2024-08-16

**Authors:** Howaida M. Hagag, Khadiga A. Ismail, Mashael M. Almutairi, Bushra I. Alnefaie, Seham H. Alajmani, Ashwaq M. Altalhi, Abdulaziz H. Alkhamash, Naif S. Althobaiti, Mohammed Awadh Alhumaidi, Ahmed Abdulwahab Bawahab, Abdulkarim Hasan

**Affiliations:** 1 Department of Clinical Laboratory Sciences, College of Applied Medical Sciences, Taif University, Taif 21944, Saudi Arabia; 2 Department of Histopathology, King Faisal Medical Complex, Taif 26514, Saudi Arabia; 3 Department of Pathology, Faculty of Medicine, University of Jeddah, Jeddah 23218, Saudi Arabia; 4 Department of Pathology, Faculty of Medicine, Al-Azhar University, Cairo 11884, Egypt; 5 Department of Laboratory, Al-Baha Health Cluster, Ministry of Health, Al-Baha 65784, Saudi Arabia

**Keywords:** chronic endometritis, high altitude, endometrial hyperplasia, D&C biopsy, CD138, abnormal uterine bleeding

## Abstract

Background: Chronic endometritis (CE) is a persistent inflammation of the uterine lining. Although it has a minimal clinical presentation, CE adversely affects the reproductive ability of women. The aims of this study were to detect pathological endometrial patterns in D&C biopsies and to evaluate chronic endometritis in patients living in a high-altitude area (1800 m above sea level) in order to determine the clinical pathological features and prevalence. Materials and methods: A cross-sectional study conducted at King Faisal Maternity Hospital included 100 samples of D&C biopsies from women complaining of various gynecological symptoms not due to gestational causes. The biopsies underwent tissue processing, H&E staining, and CD138 detection. Blood samples were taken for serological detection of infectious diseases, complete blood count, and chemical parameters. Results: The mean age of women in the study with CE was 48.5 ± 8.5 years, and that of those without CE was 46.9 ± 9.7 years. The most common complaints were abnormal uterine bleeding, accounting for 83%. CE was present in 8% of cases, and there was a nonsignificant difference in hematological parameters between women with CE and those with other pathological diagnoses. There were also nonsignificant differences in chemical parameters, except for FSH and LH levels, which showed a significant difference, with *p*-values of 0.05 and 0.02, respectively. It can be concluded that the most common gynecological complaint of women in this study was abnormal uterine bleeding. Conclusions: The most commonly diagnosed pathological endometrial disorder in D&C biopsies was disordered proliferative endometrium, followed by endometrial polyps and endometrial hyperplasia. All of these are usually associated with hormonal disturbance, which appeared to be very common in the women in this study. The prevalence of chronic endometritis detected in our study was 8%, which is relatively high.

## 1. Introduction

Endometritis is a condition characterized by inflammation of the endometrium. It can be classified into two types: acute or chronic. Acute endometritis is a symptomatic infection in the endometrium that lasts for less than 30 days. It is commonly caused by a sexually transmitted infection (STI), such as *Chlamydia trachomatis*, *Neisseria gonorrhoeae*, or bacteria associated with bacterial vaginosis (BV). The symptoms of acute endometritis are similar to those of pelvic inflammatory disease (PID) and can range from mild to severe, including fever, pelvic pain, vaginal discharge, and other signs of infection [1]. Acute endometritis is often diagnosed microscopically based on the presence of microabscesses and neutrophilic invasion in the endometrium. It is important to note that acute endometritis alone does not lead to reduced fertility rates [2]. Chronic endometritis (CE) is a silent disease that is often diagnosed during an evaluation of secondary amenorrhea (absence of menstrual periods) and infertility. Tuberculosis is a significant cause of chronic endometritis, particularly in developing nations. Both acute and chronic endometritis have been associated with poor reproductive outcomes, with worse outcomes reported for individuals with chronic endometritis [1].

Chronic nonspecific endometritis: This type of endometritis is characterized by the presence of an infiltrate of lymphocytes and plasma cells, often accompanied by a smaller component of eosinophils. It can follow pregnancy or abortion, be associated with an intrauterine device (IUD) or submucosal leiomyoma or be accompanied by mucopurulent cervicitis and/or PID. Tuberculous endometritis: Tuberculous (TB) endometritis is often focal. The appearance of a typical caseous granuloma with giant epithelioid cells is suggestive of TB. It is important to screen patients for TB by dilation and curettage or endometrial aspiration biopsy, as it can have serious health implications [3].

The prevalence of CE has been found to vary among different groups of women. In infertile women, the prevalence ranges from 2.8% to 56.8%. The prevalence of CE among women with recurrent implantation failure (RIF) is between 14% and 67.5%. Similarly, the prevalence among women with recurrent pregnancy loss ranges from 9.3% to 67.6% [4].

At high altitudes, hypoxia has a significant impact on people’s health and daily activities because it leads to profound physiological and pathological alterations in the body [5]. As evidenced by several studies, an inflammatory response can be triggered by hypoxia, and exposure at high altitudes induces cardiovascular, respiratory, and endocrine system dysfunction [6,7]. More than 130 million individuals live at high altitudes around the world. Taif is located about 1700–2500 meters above sea level, and the impact of such high altitude on global gene expression, including levels of biochemical and oxidative stress factors, must be determined in order to provide residents with the relevant information about the potential effects of hypoxia on their health [8].

The endometrium is a unique tissue that undergoes monthly cycles of change, and the effect of living at high altitudes on the endometrium is still unclear [9].

A number of clinical symptoms, including pelvic pain, dyspareunia, vaginal discharge, vaginitis, recurrent cystitis, and mild gastrointestinal discomfort, are indicative of CE, which is often asymptomatic or presents with nonspecific symptoms. The inability to pinpoint specific symptoms and the need to perform an endometrial biopsy to confirm the diagnosis make it difficult to determine the condition’s prevalence. Furthermore, because the disease progresses silently, it is often misdiagnosed or overlooked [10,11].

CE is diagnosed via endometrial biopsy using conventional hematoxylin and eosin (H&E) staining. The histopathological features of CE include superficial mucosal edema, increased endometrial stromal cell density, unsynchronized differentiation between endometrial epithelial cells and stromal fibroblasts, and abnormal invasion by lymphocytes and endometrial stromal plasmacytes (ESPCs). The hallmark feature of CE among these histological features is the presence of multiple ESPCs, which are seen in the endometrial stromal compartments as scattered or clustered cells [12].

Few studies in Saudi Arabia have investigated the morphological patterns of endometrial biopsies, and none have been conducted in a high-altitude area; therefore, this study was undertaken to examine the morphological patterns of endometrial biopsies taken from women who presented to the hospital due to infertility, uterine bleeding, and menstrual disorders. The aim is to provide an overview of the prevalence of chronic endometritis, its impact on the endometrial microenvironment, and its association with infertility.

## 2. Materials and Methods

### 2.1. Patients and Settings

A cross-sectional study was conducted from November 2022 to March 2024. Regarding the inclusion criteria, all endometrial biopsies from patients with gynecological complaints not due to gestational causes were included in the study. Relevant clinical details including age, presenting complaints, and menstrual details, including last menstrual period, periodicity, and regularity, were collected from the case records of patients. Slides stained with hematoxylin and eosin and immunohistochemically treated with CD138 were examined thoroughly, and the findings were recorded. The exclusion criteria included residing for less than 3 months in Taif, a history of tuberculous endometritis, and a lack of relevant clinical data.

### 2.2. Data and Sample Collection

A thorough history was taken for all cases in order to collect demographic and clinical information. Endometrial biopsy samples and blood samples were obtained from each patient.

### 2.3. Laboratory Investigations

Blood samples were collected for complete blood count (CBC) analysis. The collected blood samples were processed on the hematology analyzer from Sysmex with serial number 32117, which counted the number of white blood cells (WBCs) with reference range [4–10 K/μL] and red blood cells (RBCs) with reference range [3.8–4.8 M/μL]. CBC results, including the RBC count and WBC count, were generated and made available for interpretation.

Serology test reports to look for infectious diseases were analyzed using the EVOLIS device from BIO-RAD (Hercules, CA, USA) with serial number 916371170 and were obtained from the laboratory database.

Blood samples were collected for chemistry analysis. The collected blood samples were processed on the Cobas e 601 device from BIO-MED with serial number 28H3-13, which analyzed the hormonal parameters (FSH, LH, T4, T3, Prolactin).

For tissue processing and H&E staining for endometrial biopsies, H&E-stained slides were examined under the microscope, and the pathological findings of many endometrial lesions were detected, from which the finding of CE was derived (superficial mucosal edema, increased endometrial stromal cell density, unsynchronized differentiation between endometrial epithelial cells and stromal cells, and invasions by chronic inflammatory cells, with the presence of multiple endometrial stromal plasma cell (that appeared as large lymphocytes with a high nucleus/cytoplasm ratio, basophilic cytoplasm, and eccentric nuclei with heterochromatin rearrangement) as scattered cells or clustered).

### 2.4. Immunohistochemistry for CD138 on the Bond Max Machine

Cases of CE were proven via microscopic examination of H&E-stained slides, and other paraffin block sections were prepared prior for the same cases and placed onto slides with a positive charge for staining using a CD138 detection kit to detect stromal plasma cells. The slides were covered with a cover slip after being applied with the CD138 detection kit. The slides were stained using the device (The Bond Max machine from Leica (Wetzlar, Germany), with serial number M495558), and the results were interpreted automatically using the machine software after comparing the positive and negative control slides. A positive result is indicated when a brown color is observed on the plasma cell membrane, indicating the existence of plasma cells. On the other hand, a blue color (absence of brown color) was seen as a negative reaction, indicating the presence of other inflammatory cells and the absence of plasma cells.

### 2.5. Sample Size Estimation

A sample size of 92 cases achieves 80% power to reject the null hypothesis of a zero-effect size when the population effect size is 0.8, and the significance level (Alpha) is 0.05 using a two-sided two-sample equal-variance *t*-test. However, the number was increased to 100 to show appropriate results and improve the study’s strength.

### 2.6. Data Processing and Statistical Analysis

SPSS software (version 25; IBM Crop, Armonk, NY, USA) was used to conduct statistical analysis. In the quantitative data, the mean, SD, and range were displayed. Frequency and percentage were used to represent the qualitative data. At the 5% level, the significance of the results was assessed. This study employed a chi-squared test (χ^2^) to examine the correlation between qualitative variables. When more than 20% of the cells have an anticipated count of less than 5, Fisher’s Exact or Monte Carlo adjustment is used for chi-square analysis. When comparing quantitative variables between two sets of normally distributed data, Student’s T test (t) was utilized. Quantitative variables that are not normally distributed between two data groups are compared using the Mann–Whitney U test (U). When the *p*-value was less than 0.05, it was regarded as statistically significant.

## 3. Results

**Figure 1:**
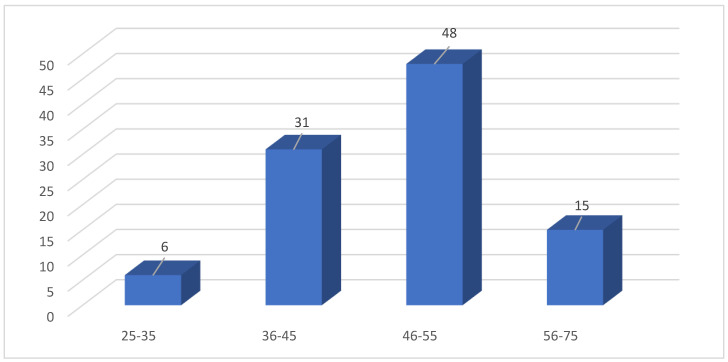
Age group (in years) distribution of females under study.

This study included a total of 100 patients, and the age of the included cases ranged from 25 to 75 years; their mean age ± standard deviation (SD) is 48.5 ± 8.5 in cases with CE and 46.9 ± 9.7 in cases without CE, arranged in four age groups, as shown in Figure 1. The age group most likely to suffer from gynecological manifestations and undergo D&C biopsy was the 46–55 age group, accounting for 48% of cases, followed by the 36–45 age group, representing 31% of cases, and the least likely age group was 25–35.

The most common gynecological complaint was abnormal uterine bleeding, accounting for (83/100, 83%) of cases. This is followed by pelvic pain, which presented in (48/100, 48%) of cases. The percentage of cases seeking pregnancy was 5%, while the percentage of cases complaining of swelling was (4/100, 4%).

**Figure 2:**
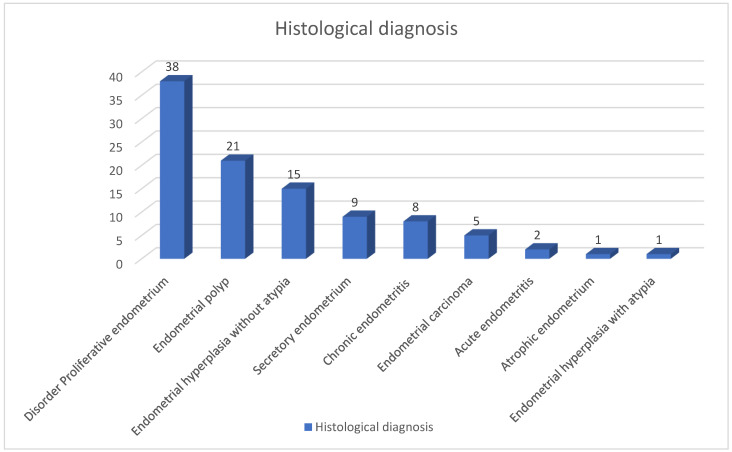
Histopathological diagnosis of endometrial biopsies of the females under study.

With regard to the prevalence of histopathological diagnoses based on D&C biopsies of the women in this study, the most common diagnosis was disordered proliferative endometrium, accounting for 38% of cases, followed by endometrial polyps, present in 21% of cases, endometrial hyperplasia, present in 15%, secretory endometrium, present in 9%, CE present in 8%, endometrial carcinoma, present in 5%, and endometrial hyperplasia with atypia present in 1% of cases, as shown in Figure 2.

With regard to the prevalence of different histopathological diagnoses of D&C biopsies for included cases, the most common diagnosis was disordered proliferative endometrium, accounting for 38% of cases, followed by endometrial polyp, representing 21% of cases. Endometrial hyperplasia without atypia was present in 15% of cases; secretory endometrium represents 9%, while the cases with CE accounted for 8% of cases. Endometrial carcinoma was present in 5%, and the least diagnosis was endometrial hyperplasia with atypia, which was present in 1% of cases.

**Figure 3:**
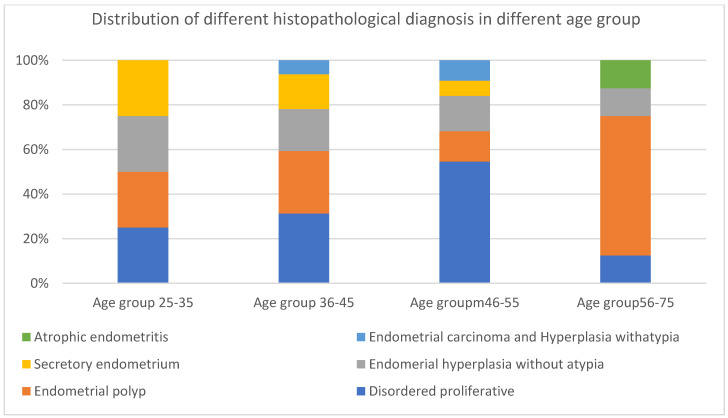
Histological diagnosis in different age groups of females under study.

Regarding the distribution of different histopathological diagnoses based on D&C biopsies in different age groups, most cases suffering from disordered proliferative endometrium, endometrial polyps, chronic endometritis, and endometrial hyperplasia without atypia were all in the 46–55 year age group. Most cases suffering from secretory endometrium were in the 36–45 year age group. Most cases suffering from endometrial carcinoma and cases with atrophic endometrium were in the 56–75-year age group, as shown in Table 1. The mean age and standard deviation in cases with CE was 46.9 ± 9.7, and the mean age and standard deviation in cases with other pathological diagnoses was 48.5 ± 8.5, with non-statistically significant differences, as shown in Table 1 and Figure 3.

**Figure 4:**
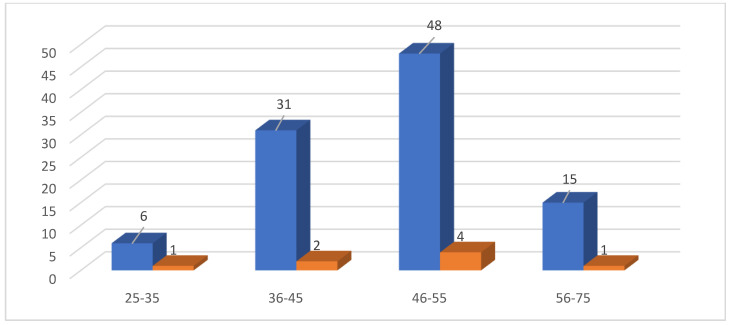
Distribution of cases with chronic endometritis among different age groups (blue column: D&C biopsies. Brown column: CE cases).

Regarding the distribution of cases with chronic endometritis in different age groups, there were four cases (50%) present in the 46–55 year age group, and two (25%) cases present in the 36–45 year age group. The 25–35 and 56–75 age group each contain one case of chronic endometritis, as shown in Figure 4.

Regarding the correlation between age, hematological, and chemical parameters in cases of chronic endometritis and cases with other histopathological diagnosis, only two cases had infections, one by *Cytomegalovirus* (CMV), and the other by human immunodeficiency virus (HIV); these were not associated with chronic endometritis cases. There was a nonsignificant difference between the hematological parameters in cases with CE and cases with another diagnosis. Regarding the chemical parameters (prolactin, T3, and T4 hormonal levels), there was also a nonsignificant difference; however, there was a significant difference in LH and FSH levels, with *p* values of 0.05 and 0.02, respectively, as shown in Table 2.

Moreover, there was no significant association between CE cases and infertility.

**Figure 5:**
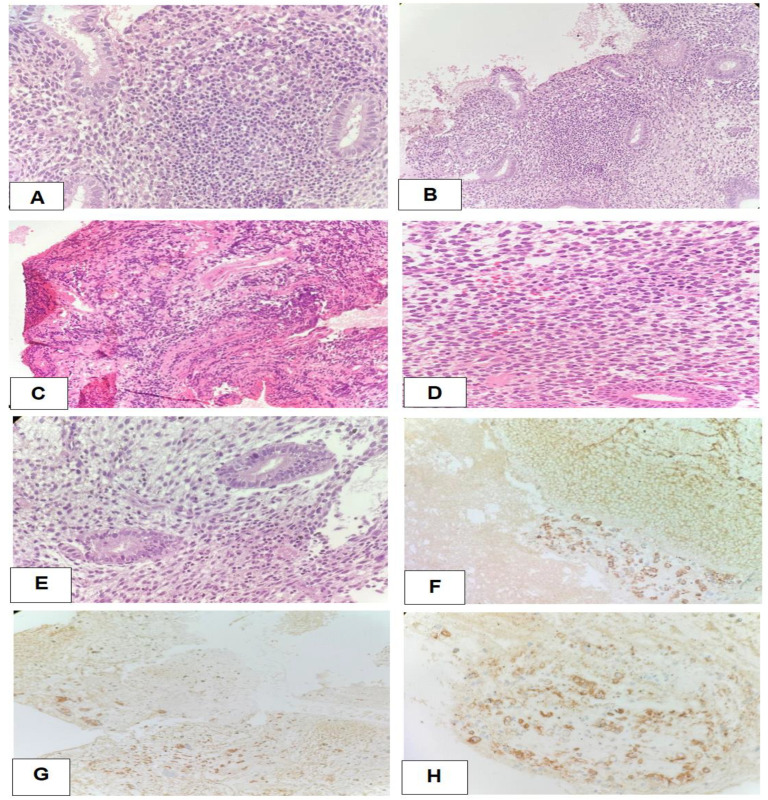
(**A**–**H**) Photomicrograph of 4 microns thick H&E-stained paraffin sections and CD138 immunohistochemically stained sections. (**A**,**B**): The examined microscopic sections from endometrial curette biopsies showing dense endometrial stroma infiltrated by chronic inflammatory cells with scattered plasma cells (original magnification ×20–×10). (**C**,**E**): Chronic endometritis cases showing endometrial stromal fibrosis and chronic inflammatory infiltrate with clustered plasma cells (original magnifications ×20–×10). (**D**): Endometrial stromal edema and lymphoplasmacytic infiltration (original magnification ×20). (**F**–**H**): The stromal plasma cell membrane immunoreactivity by CD138 brown staining (original magnifications ×40–×20–×40).

All cases of CE showed positive staining of CD138 immunohistochemical marker that confirm the mature of plasma cells (Figure 5).

## 4. Discussion

Normally, during the menstrual cycle, the endometrium is infiltrated by immunocompetent cells, such as natural killer cells, macrophages, and T cells. The cyclic changes affect tissue remodeling, making the endometrium receptive. Cycle-dependent changes in leukocyte subpopulations and their mediators likely play essential roles in implantation. On the other hand, in normal human endometrium, B cells constitute less than 1% of the total number of leukocytes. Endometrial B cells are rarely seen in the functional layer (the part that is shed during menstruation) but are found in the basal layer (the part that remains throughout the menstrual cycle) as central cells in the lymphocyte population surrounded by numerous CD8+ T cells and macrophages [1,2,13]. The uterus is usually sterile, but the migration of microbes from the nearby lower genital tract causes inflammation and changes in the endometrium, such as edema and plasma cell infiltration in the stroma, potentially impacting endometrial receptivity. A bacterial infection in the endometrium induces proinflammatory cytokines and chemokines in the endometrial cells, leading to the extravasation and migration of a large number of circulating B lymphocytes into the endometrial tissue, glandular epithelium, and lumina.

The abnormal expression of multiple proinflammatory molecules involved in B cell extravasation into endometrium with CE has been demonstrated. The adhesion molecule selectin E, which is essential for the attachment and rolling of circulating B lymphocytes on endothelial cells, is expressed by endometrial microvascular endothelial cells in the case of CE, whereas selectin E is not found in non-pathologic human endometrium. CXCL13, a chemoattractant that activates tight adhesion molecules on B cells and endothelial cells, is also expressed by endometrial microvascular endothelial cells in cases of CE. Furthermore, the chemokine CXCL1, which plays a role in B cell migration, is aberrantly expressed by endometrial glandular epithelial cells. Some accumulated endometrial B lymphocytes are able to turn into endometrial stromal plasmacytes (ESPCs) in situ, with CD138 (+) serving as the marker. It has been observed that ESPCs express several immunoglobulin subclasses, including IgM, IgA1, IgA2, IgG1, and IgG2, with the majority being IgG2. Elevated levels of these mucosal antibodies likely have a negative impact on the embryo implantation process as they lead to defects in endometrial receptivity as a result of being filled with antibodies and inflammatory cells [10,11,12,13,14].

Chronic endometritis (CE) is a persistent inflammation characterized by infiltration of endometrial stroma by plasma cells with other chronic inflammatory infiltrates. It is often asymptomatic or has nonspecific symptoms. However, it can also produce lower pain in the abdomen, abnormal uterine bleeding, and increased secretions from the vagina. CE can also impact endometrium receptivity, leading to recurrent miscarriages, infertility, and unsuccessful embryo implantation [15].

This study included 100 patients who were living at Taif and who were admitted to King Faisal Medical Complex maternity hospital between November 2022 and March 2024. Their ages range from 25 to 75 years, and (48/100, 48%) of the cases were observed in the age group between 46 and 55 years. The lowest percentage was found in the age group between 25 and 35, as shown in Figure 1.

Our results agree with the findings obtained by Sajitha et al. [16], where the highest prevalence of gynecological conditions, including abnormal uterine bleeding (AUB), was observed in the 46–55 age group, particularly in relation to D&C biopsies. This age range coincides with the perimenopausal age group, which is characterized by significant hormonal fluctuations, particularly a decline in estrogen levels. This finding suggests a potential link between perimenopausal hormonal changes and an increased risk of gynecological conditions. Further research is warranted to explore the specific hormonal mechanisms influencing the prevalence of AUB during this critical stage in a woman’s reproductive health.

Our study demonstrated that the most common gynecological complaint was abnormal uterine bleeding, accounting for (83/100, 83%) of cases. This is followed by pelvic pain, which presented in (48/100, 48%) of cases, which may be caused by hormonal disturbances. The percentage of cases seeking pregnancy was 5%, while the percentage of cases complaining of swelling was (4/100,4%). A research study conducted by MacLean et al. [17] explains that the imbalance of estrogen and progesterone disrupts their complex regulatory mechanisms, leading to estrogen dominance and progesterone resistance. These hormonal imbalances can contribute to gynecological manifestations, including abnormal uterine bleeding and pelvic pain.

In our study, as shown in Figure 2 the most common histopathological diagnosis for D&C biopsies carried out for the women under study was disordered proliferative endometrium (38/100, 38%), followed by endometrial polyps (21/100, 21%) and endometrial hyperplasia without atypia (15/100, 15%). This was not consistent with a study conducted by Sushma et al. [18], which showed that out of 72 analyzed biopsies, disordered proliferative endometrium was present in 13.88% of cases, endometrial polyps in 2.77%, and endometrial hyperplasia without atypia in 5.55% of cases. This difference could be due to geographical variations and the selective collection of biopsies from women who reported abnormal uterine bleeding.

The prevalence of chronic endometritis in our study, was 8 out of 100 analyzed biopsies, while in a study conducted by Song et al. [19], the prevalence rate of CE was found to be 24.4% within 1551 analyzed biopsies, which is higher and not consistent with the findings of our study. This might be due to the significant difference in the number of analyzed biopsies and in the duration of the study. In another study conducted by Husain et al. [20], they found that the prevalence of CE was 10.3% of the analyzed biopsies; this is consistent with the findings of our study.

Due to the low prevalence rate of CE in this study as a result of the low sample size, as well as the fact that all endometrial biopsies from women who presented to the department of obstetrics and gynecology were taken without allocating biopsies to women with reproductive problems and infertility, the association of infertility with CE was not adequately determined.

In this study, as shown in Table 1 and Figure 2, most cases suffering from disordered proliferative endometrium and endometrial polyps were all in the 46–55 year age group, while most cases suffering from endometrial carcinoma and cases with atrophic endometrium were in the 56–75 years age group, which is consistent with a study conducted by Husain et al. [20] that found that disordered proliferative endometrium and endometrial polyps are most common in the 40–55 years age group, while endometrial carcinoma and atrophic endometrium were most common in the over-55 age group. However, there was one contrast in the age group of endometrial hyperplasia without atypia. In our study, most cases were in the 46–55 age group, while in the study conducted by Husain et al. [20], it was most common in the over-55 age group. This difference might be attributed to racial and ecological factors.

Figure 4 shows that, in this study, most cases, 50%, with chronic endometritis were present in the 46–55 years age group, and 25% of cases presented in the 36–45 years age group, which was in not consistent with a study conducted by Jain and Jain [3] that found most CE cases to be in the 25–35 years age group. This discrepancy may be due to differences in the geographic regions and the prevalence of gynecological infection.

In another study conducted by Husain et al. [20], they found most cases with chronic endometritis to be in the 40–55 years age group, and this was in agreement with the findings of our study.

In Table 2, no significant changes in hematological and hormonal parameters can be seen, either in CE cases or in non-CE cases, except for the statistically significant increase in the levels of FSH and LH, which are mostly seen in perimenopausal cases; this may be due to the hormonal disturbance that occurs in them at this stage and because most of the biopsies were taken from older women.

Plasma cells, the end point of B cell differentiation, represent the main component in chronic nonspecific endometritis. As terminally differentiated cells, plasma cells remain quiescent and unable to proliferate; their generation occurs, and could increase, under hypoxic conditions [21].

However, hypoxia increases the generation of plasma blasts, starting from memory B cells via increasing the cell cycle and division number. Its role in CE is still unclear and information has not been provided in the literature. This study tried to shed light on the status of the endometrium in high-altitude cases but could not assess the status of hypoxia.

None of the chronic endometritis cases in this study were associated with infertility or infection, and these findings contrasted with the results of a study conducted by Giraldo-Isaza et al. [22], who found that endometritis was secondary to herpes simplex virus (HSV) and cytomegalovirus (CMV) infections, particularly in patients with HIV. The causes of the absent association between CE and infertility can explained by the fact that the majority of cases were of premenopausal age and the fact that most cases came to the hospital for complaints other than infertility or pregnancy problems. Furthermore, the absence of positivity in cases of chronic endometritis to serological tests carried out to detect some viral infection could be due to the fact that most cases of chronic endometritis under study were old and thus not exposed to risk of infections relating to the use of intrauterine devices or labor.

Chronic endometritis has minimal clinical manifestations but has adverse effects on the reproductive function of the uterus. This adverse effect was not detected in our study due to some limitations.

### Limitation of the Study

Most women under study were of premenopausal age; therefore, they came to the hospital and underwent D&C biopsies for complaints other than pregnancy problems. This study included all cases that underwent D&C biopsies for any gynecological manifestation, not only those with infertility or reproductive problems; therefore, the relation between CE and female fertility may not have been properly detected. Based on the prevalence rates of endometrial pathology detected in our study, we recommend the importance of national programs for females that raise awareness about the risks of hormonal disturbances in the development of most reproductive system diseases and gynecological manifestations, providing further guidance on avoiding the causes of this hormonal imbalance, such as obesity, bad dietary habits, and unjustified hormonal intake. A future long-term study should be conducted in medical centers specializing in infertility on a large number of cases complaining of infertility or failure to conceive.

## 5. Conclusions

The prevalence of chronic endometritis detected in our study was 8%, which is relatively not unusual, and no significant association between CE cases and infertility was found. The most common gynecological indication for D&C found in this study was abnormal uterine bleeding that caused patients to seek medical advice and histopathological diagnosis. The most commonly diagnosed pathological endometrial disorder found in the D&C biopsies was disordered proliferative endometrium, followed by endometrial hyperplasia.

## Figures and Tables

**Table 1:** Histological diagnosis in different age groups of females under study.

Histological Diagnosis	Age Groups				χ^2^	*p* Value
	25–35 *n* = 6 (%)	36–45 *n* = 31 (%)	46–55 *n* = 48 (%)	56–75 *n* = 15 (%)		
Disorderd proliferative endometrium	1 (17%)	10 (32%)	24 * (50%)	3 (20%)	5.6	0.017 ^#^
Secretory endometrium	1 (17%)	5 ** (16%)	3 (6%)	0 (0%)	7.1	0.007 ^#^
Atrophic endometrium	0 (0%)	0 (0%)	0 (0%)	1 *** (7%)	5.7	0.016 ^#^
Endometrial polyp	1 (17%)	6 (19%)	9 (19%)	5 (33%)	0.2	0.595
Chronic endometritis	1 (17%)	2 (6%)	4 (8%)	1 (7%)	0.01	0.906
Acute endometritis	1 (17%)	0 (0%)	1 (2%)	0 (0%)	0.003	0.954
Hyperplasia without atypia	1 (17%)	6 (19%)	7 (15%)	1 (7%)	0.01	0.911
Hyperplasia with atypia	0	1 (3%)	0 (0%)	0 (0%)	2.2	0.133
Endometrial carcinoma	0	1 (3%)	0 (0%)	4 (27%)	1.7	2.96

* Significant association of disordered proliferative endometrium in 46–55 age group; ** Significant association of secretory endometrium in 36–45 age group; *** Significant association of atrophic endometrium in 56–75 age group; ^#^ Statistically significant.

**Table 2:** Age, hematological, and chemical parameters in cases with and without chronic endometritis.

	Cases with Chronic Endometritis		Cases without Chronic Endometritis		*t*-Test	*p* Value	Sig
	Mean	±SD	Mean	±SD			
Age	46.9	9.7	48.5	8.5	0.4	0.8	NS *
WBC	7.7	2.3	6.8	2.3	1.02	0.30	NS
RBC	4.4	0.49	4.6	0.49	1.03	0.70	NS
LH	24.1	6.1	11.9	2.2	1.9	0.05	S ^#^
FSH	40.2	17.2	20.2	3.5	1.72	0.02	S ^#^
Prolactin	157	20.1	302	31.6	0.76	0.4	NS
T3	4.7	0.49	4.2	0.87	1.16	0.2	NS
T4	12.9	4.9	14.3	2.2	1.31	0.1	NS

^#^ S: statistically significant. * NS: non-statistically significant.

## Data Availability

The data presented in this study are available on request from the corresponding author.
